# Author Correction: Enhancer hijacking at the *ARHGAP36* locus is associated with connective tissue to bone transformation

**DOI:** 10.1038/s41467-023-42123-7

**Published:** 2023-10-09

**Authors:** Uirá Souto Melo, Jerome Jatzlau, Cesar A. Prada-Medina, Elisabetta Flex, Sunhild Hartmann, Salaheddine Ali, Robert Schöpflin, Laura Bernardini, Andrea Ciolfi, M-Hossein Moeinzadeh, Marius-Konstantin Klever, Aybuge Altay, Pedro Vallecillo-García, Giovanna Carpentieri, Massimo Delledonne, Melanie-Jasmin Ort, Marko Schwestka, Giovanni Battista Ferrero, Marco Tartaglia, Alfredo Brusco, Manfred Gossen, Dirk Strunk, Sven Geißler, Stefan Mundlos, Sigmar Stricker, Petra Knaus, Elisa Giorgio, Malte Spielmann

**Affiliations:** 1 Max Planck Institute for Molecular Genetics, Development and Disease Group, 14195 Berlin, Germany; 2 Institute for Medical Genetics and Human Genetics, Charité University Medicine Berlin, 13353 Berlin, Germany; 3 Freie Universität Berlin, Institute for Chemistry and Biochemistry, 14195 Berlin, Germany; 4 Istituto Superiore di Sanità, Department of Oncology and Molecular Medicine, 00161 Rome, Italy; 5 Cytogenetics Unit, Casa Sollievo della Sofferenza Foundation, IRCCS, 71013 San Giovanni Rotondo, Foggia Italy; 6 Molecular Genetics and Functional Genomics, Ospedale Pediatrico Bambino Gesù, IRCCS, 00146 Rome, Italy; 7 Max Planck Institute for Molecular Genetics, Department of Computational Molecular Biology, 14195 Berlin, Germany; 8 Department of Biotechnology, University of Verona, 37129 Verona, Italy; 9 Julius Wolff Institute (JWI), Berlin Institute of Health at Charité – Universitätsmedizin Berlin, 13353 Berlin, Germany; 10 BIH Center for Regenerative Therapies (BCRT), Berlin Institute of Health at Charité – Universitätsmedizin Berlin, 10117 Berlin, Germany; 11 Institute of Active Polymers, Helmholtz-Zentrum Hereon, 14513 Teltow, Germany; 12 Berlin-Brandenburg Center for Regenerative Therapies (BCRT), 13353 Berlin, Germany; 13 Department of Clinical and Biological Sciences, University of Torino, 10043 Torino, Italy; 14 Department of Medical Sciences, University of Torino, 10126 Torino, Italy; 15 Medical Genetics Unit, Città della Salute e della Scienza University Hospital, Torino, 10126 Italy; 16 Cell Therapy Institute, Spinal Cord Injury and Tissue Regeneration Center Salzburg (SCI-TReCS), Paracelsus Medical University (PMU), 5020 Salzburg, Austria; 17 Department of Molecular Medicine, University of Pavia, 27100 Pavia, Italy; 18 Medical Genetics Unit, IRCCS Mondino Foundation, 27100 Pavia, Italy; 19 Institute of Human Genetics, University Hospitals Schleswig-Holstein, University of Lübeck and University of Kiel, Lübeck, 23562 Germany; 20 DZHK (German Centre for Cardiovascular Research) Germany, partner site Hamburg, Lübeck, Kiel, Lübeck, 23562 Germany

**Keywords:** Gene expression, Disease genetics, Chromatin structure

Correction to: *Nature Communications* 10.1038/s41467-023-37585-8, published online 11 April 2023

In the Acknowledgements section of this article, one of the funding sources was incorrect.

The following paragraph: “This study was also supported by the Baylor-Hopkins Center for Mendelian Genomics through National Human Genome Research Institute grant 5U54HG006542 (genome sequencing), Ministero dell’Istruzione, dell’Università e della Ricerca—MIUR “Dipartimenti di Eccellenza 2018–2022” to Department of Medical Sciences, University of Torino (Project no. D15D18000410001), and Italian Ministry of Health (5 × 1000), Fondazione Bambino Gesù (Vite Coraggiose) to M.T.”

was replaced with: “Genome sequencing was provided by the University of Washington Center for Mendelian Genomics (UW-CMG) and was funded by NHGRI and NHLBI grants UM1 HG006493 and U24 HG008956. This study was also supported by Ministero dell’Istruzione, dell’Università e della Ricerca—MIUR “Dipartimenti di Eccellenza 2018–2022” to Department of Medical Sciences, University of Torino (Project no. D15D18000410001), and Italian Ministry of Health (5 × 1000), Fondazione Bambino Gesù (Vite Coraggiose) to M.T.”

The original article has been corrected.

