# Supplementary material for: Enhancer hijacking at the ARHGAP36 locus is associated with connective tissue to bone transformation
Source: Nat Commun. 2023 Apr 11;14:2034. doi: 10.1038/s41467-023-37585-8 (PMC10090176; doi:10.1038/s41467-023-37585-8)
Supplement: Supplementary file 1 — Supplementary Information [file 41467_2023_37585_MOESM1_ESM.pdf]

Supplementary Information

Duplicated genes excluded as candidate to explain the phenotype

The 820 kb duplication piggybacked six protein coding genes into chrX. Half of them (*GFPT1*, *NFU1* and *AAK1*) were overexpressed, while the other half (*ANXA4*, *GMCL1* and *SNRNP27*) were not. Here we provide a comprehensive explanation of the rationale, although speculative, behind discarding these genes as causatives for the disease. First, using the fibroblast RNA-seq data, we excluded, as expected, gene-fusions involving *ANTXR1* or *MXD1* (data not shown). Next, we evaluated if the abovementioned six genes are associated with bone formation pathways, such as BMP-TGFB, Wnt and HH; and/or are associated with genetic disorders that have symptoms overlapping with bone formation. Based on these two criteria, we analyzed each of the six protein-coding genes as candidates:

- *GFPT1* encodes for an enzyme that controls glucose flux into the hexosamine pathway and catalyzes the formation of glucose 6-phosphate. This gene has been previously associated with myasthenic syndrome, congenital 12, with tubular aggregates (MIM 610542), an autosomal recessive condition characterized by proximal muscle weakness in the first decade of life. Around 20 missense and loss-of-function mutations have been associated with this disease, and most of the patients' samples studies showed reduced amounts of the GFPT1 protein [76,77]. On the grounds that loss-of-function *GFPT1* variants cause myasthenic syndrome in biallelic state, a disease with no overlap with heterotopic ossification, we excluded this candidate to explain the proband phenotype.
- *NFU1* encodes for a protein involved in iron-sulfur cluster biogenesis. Biallelic loss-of-function mutations in this gene lead to multiple mitochondrial dysfunction syndrome, an autosomal recessive disease. NFU1 protein was not detected in patients' samples from published studies, whereas in our proband this gene is overexpressed. This syndrome's phenotypic traits are very severe and not consistent with the phenotype of the proband in this study.

- *AAK1* (AP2 Associated Protein Kinase 1) encodes for a Ser/thr protein kinase that triggers clathrin assembly during receptor mediated endocytosis. It phosphorylates a subunit of the AP2 complex, subsequently leading to receptor endocytosis. No disease has been associated so far with this gene and overlapping duplications containing this gene, plus several others, in Decipher, showed no overlap of phenotypes.
- *ANX4* - Annexin IV (ANX4) belongs to the annexin family of calcium-dependent phospholipid binding proteins. Although their functions are still not clearly defined, several members of the annexin family have been implicated in membrane-related events along exocytotic and endocytotic pathways. No disease has been associated so far with this gene.
- *GMCL1* - This gene encodes a nuclear envelope protein that appears to be involved in spermatogenesis, either directly or by influencing genes that play a more direct role in the process. This multi-exon locus is the homolog of the mouse and *Drosophila* germ cell-less gene but the human genome also contains a single-exon locus on chromosome 5 that contains an open reading frame capable of encoding a highly-related protein. No disease has been associated so far with this gene.
- *SNRNP27* - This gene encodes a serine/arginine-rich (SR) protein. SR proteins play important roles in pre-mRNA splicing by facilitating the recognition and selection of splice sites. No disease has been associated so far with this gene.

Copy number variation (CNV) analysis using decipher database (https://www.deciphergenomics.org/) showed few duplications overlapping these genes, which are, however, long Mb duplications, containing several genes, and showing syndromic features not observed in the studied proband. Taking together, we consider it very unlikely that these genes may be causative for the mentioned disease.

Inheritance of the de novo autosomal duplication and X-chromosome inactivation

Autosomal segments translocated to chromosome X in female mammalians can cause skewed X chromosome inactivation, therefore silencing of inserted autosomal genes and the derivative X [78]. Here we sought to investigate the status of X inactivation in our proband after the chromosome 2 fragment being inserted on Xq26.1. First, leveraging haplotype information from trio genome sequencing, we used phased-haplotype sequences from three regions of chromosomes 2 (upstream, 820 kb duplication and downstream) and counted the number of reads inherited from the maternal and paternal alleles. Upstream and downstream regions showed a similar number of maternal and paternal reads in the proband (Figure S3A). On the other hand, the 820 kb region in the proband showed an increased number of maternal reads, indicating that the maternal haplotype sequence was used as a template for the de novo duplicated fragment. Next, haplotype-phased RNA-seq data from the proband revealed a roughly 70:30 of maternal/paternal expression ratio throughout active genes on chrX, therefore suggesting that der(X) is undergoing less X inactivation, at least in fibroblast samples.

References

76 Senderek, J. *et al.* Hexosamine biosynthetic pathway mutations cause neuromuscular transmission defect. *The American Journal of Human Genetics* **88**, 162-172 (2011).

77 Helman, G. *et al.* Leukoencephalopathy due to variants in GFPT1-associated congenital myasthenic syndrome. *Neurology* **92**, e587-e593 (2019).

78 Allshire, R. C. & Madhani, H. D. Ten principles of heterochromatin formation and function. *Nature reviews Molecular cell biology* **19**, 229-244 (2018).

Supplementary Table

Table S1 . List of genes associated with fibrodysplasia ossificans progressiva (FOP), heterotopic ossification (HO) or ectopic calcification in the literature. Legend: AD - autosomal dominant; AR - autosomal recessive

Phenotype	Genes	OMIM #	Inheritance	Variant model	Reference
Pseudoxanthoma elasticum	ABCC6	# 264800	AD, AR	?	Glatz, A. C., et al. 2006.
Fibrodysplasia ossificans progressiva	ACVR1	# 135100	AD	Gain-of-function	Shore, E. M., et al. 2006.
Hypocalciuric hypercalcemia, type I	CASR	# 145980	AD	Loss-of-function	Pollak, M. R., et al. 1993.
Arterial calcification, generalized, of infancy, 1	ENPP1	# 208000	AR	Loss-of-function	Rutsch, F., et al. 2001.
Tumoral calcinosis, hyperphosphatemic, familial, 2	FGF23	# 211900	AR?	Loss-of-function?	Benet-Pages, A., et al. 2005.
Tumoral calcinosis, hyperphosphatemic, familial, 1	GALNT3	# 211900	AR	Loss-of-function	Topaz, O., et al. 2004.
Osseous heteroplasia, progressive	GNAS	# 166350	AD	Loss-of-function?	Shore, E. M., et al. 2002.
Tumoral calcinosis, hyperphosphatemic, familial, 3	KL	# 617994	AR	Loss-of-function	Ichikawa, S., et al. 2007.
Tumoral calcinosis, familial, normophosphatemic	SAMD9	# 610455	AR	Loss-of-function	Topaz, O., et al. 2006.

Supplementary Figures

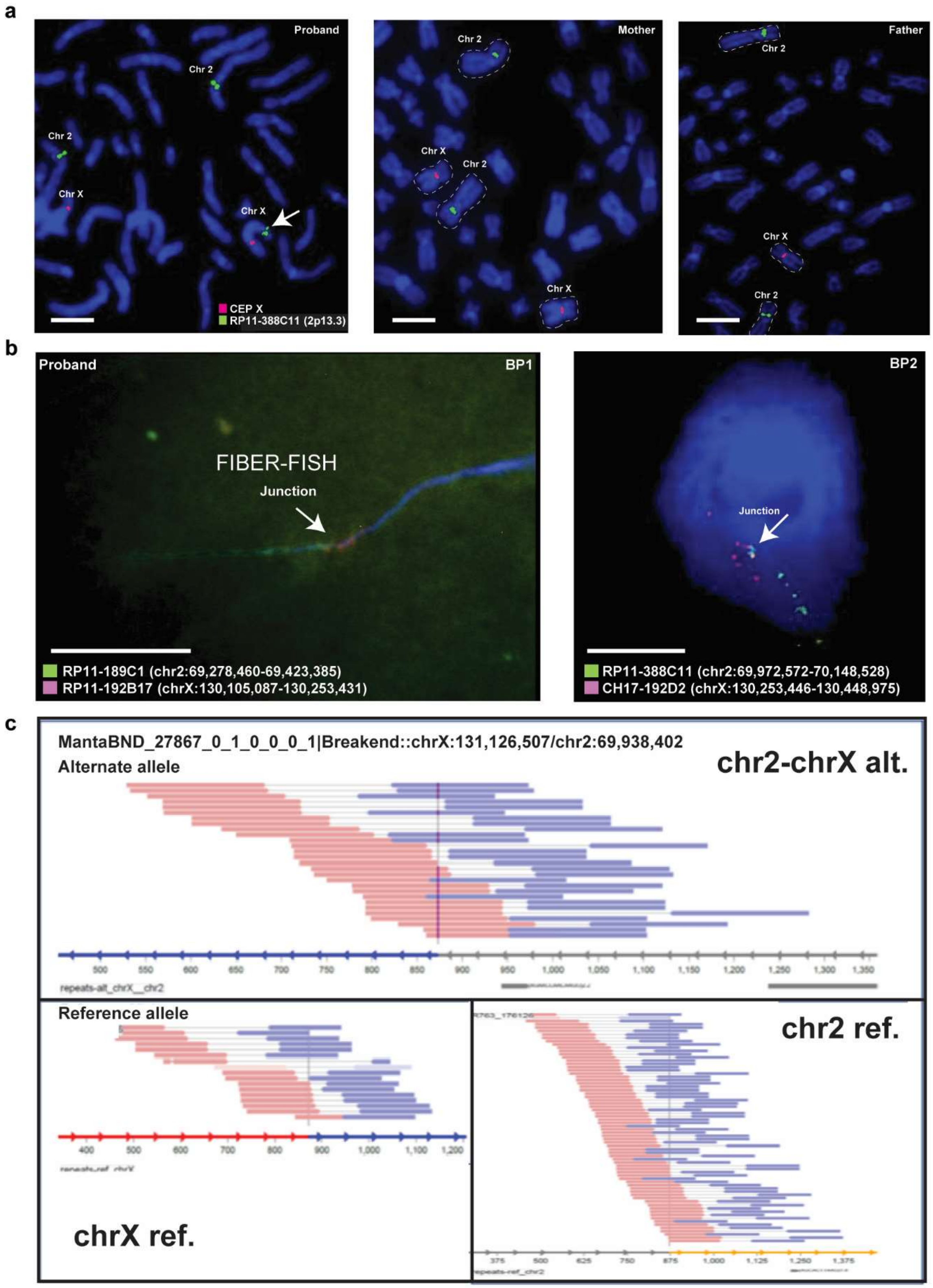

Figure S1. Fluorescence in situ hybridization (FISH) and genome sequencing. (a) FISH analysis using BAC probes overlapping the *MXD1* gene showed that the *de novo* duplicated segment

[der(X)ins(X;2)(q26.1;p13.3)] was inserted on Xq26.1. Scale bar = 5uM. (b) FIBER-FISH detected the junction point between chr2-chrX. (c) Genome sequencing detected the duplicated fragment GRCh37/hg19; chr2:69,343,058-70,165,537 and shows several read pairs spanning chr2-chrX region at chrX:130,259,064-130,260,484 (GRCh37/hg19).

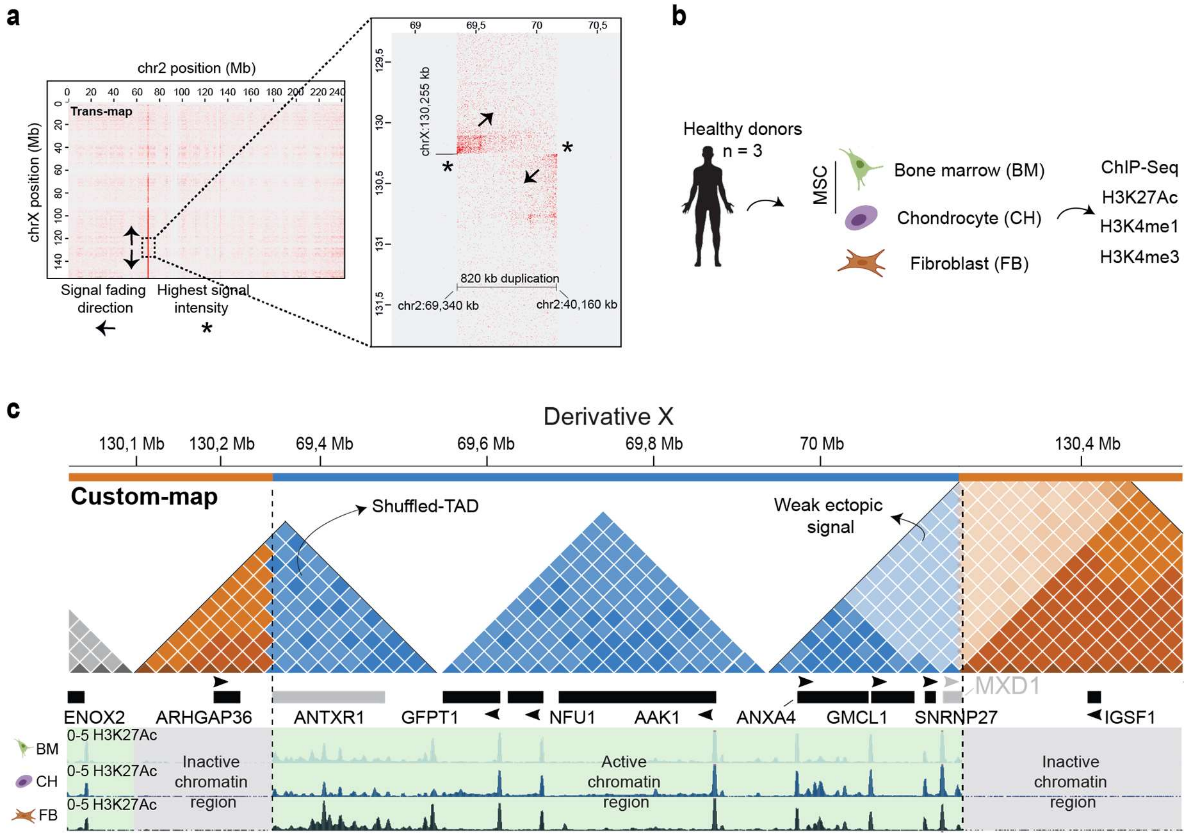

Figure S2. Chromatin landscape in wild type MSCs and fibroblasts. (a) Left panel: Trans-Hi-C map shows bidirectional ectopic chromatin signal all over the chrX, fading from the insertion breakpoint towards both telomeres. Right panel: Zoom in to the region with highest signal intensity shows the signal fading direction (black arrows), indicating that the duplicated 820 kb was inserted in the sense orientation. Note that the Hi-C reads observed here are later remapped onto the customized der(X) (Figure 2c). (b) Schematics of the ChIP-seq dataset for H3K4me1, H3K4me3 and H3K27ac epigenetic marks in two different mesenchymal stromal cell (MSC) and fibroblasts from healthy donors (bone marrow, BM; chondrocyte, CH; and fibroblast; FB). (c) Two regimes of chromatin status are observed in wild type MSCs and fibroblasts: the chr2 locus contains several active epigenetic marks (active chromatin domain; highlighted in light green), whereas the *ARHGAP36* and *IGSF1* TAD on chrX shows no epigenetic signal (inactive chromatin domain; light grey).

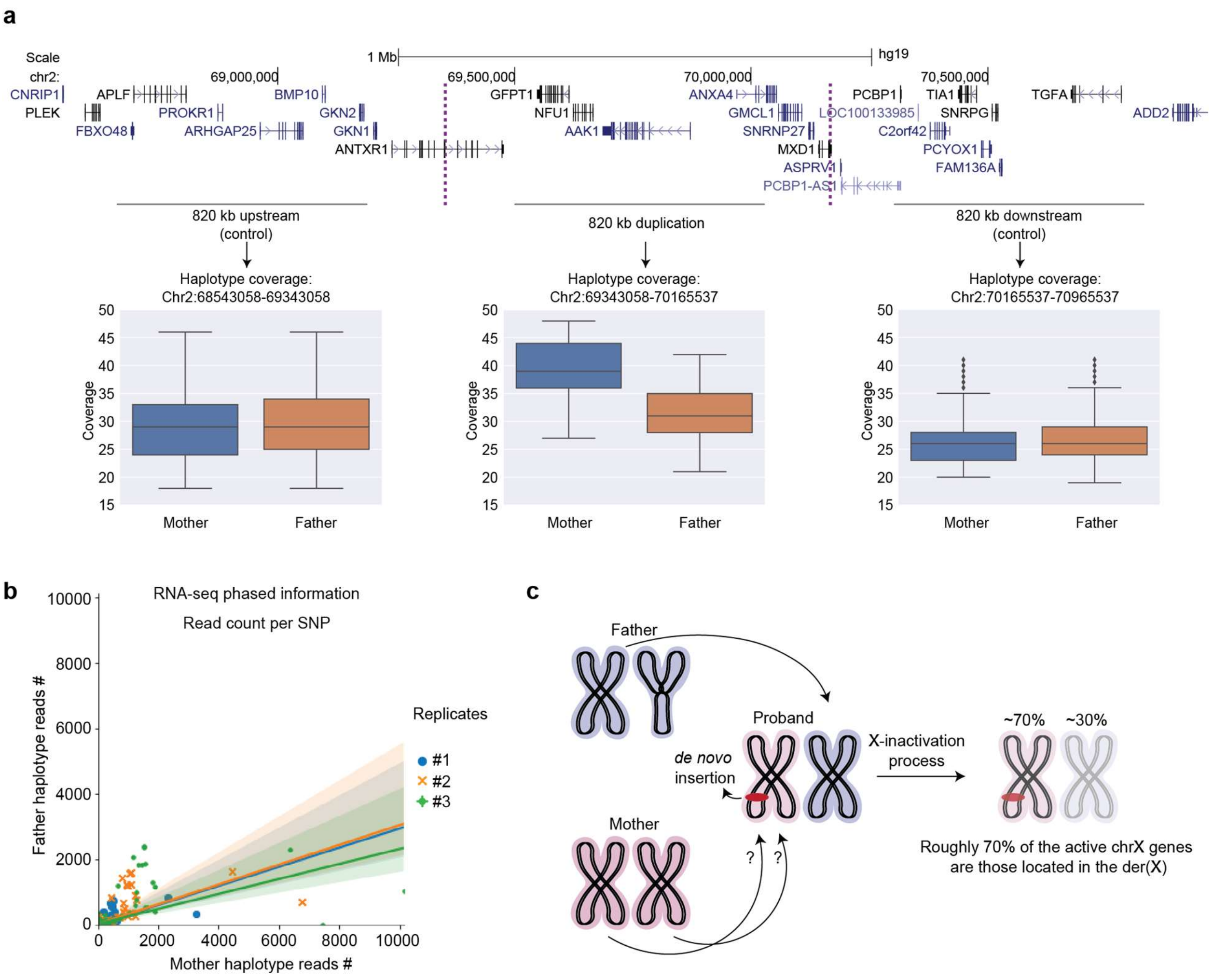

Figure S3. Inheritance of the *de novo* autosomal duplication and X-chromosome inactivation. (a) Reads coverage analysis of the proband GS data on phased variants (maternally inherited in blue; paternally in orange). Haplotype-based analysis indicated the maternal allele was used as a template for the *de novo* duplicated fragment. The median score is represented by the horizontal line in the center. The 25th and 75th percentile values are indicated by the lower and upper limits of the box. Additionally, the box plot displays outliers marked with circles. The higher and lower whiskers show the observed values that are not considered outliers. (b) RNA-Seq coverage analysis of the phased variants on chrX. Each color indicates a replicate and each dot is the RNA-Seq coverage of the variants with known parental origin. RNA-seq haplotype-phased data revealed roughly 70:30 maternal/paternal expression ratio throughout active genes on chrX. The translucent band around the regression line shows the confidence interval for each replicate estimated using bootstrap. (c) Schematic representation of X inactivation seen in the proband fibroblasts. The der(X) is not undergoing X inactivation.

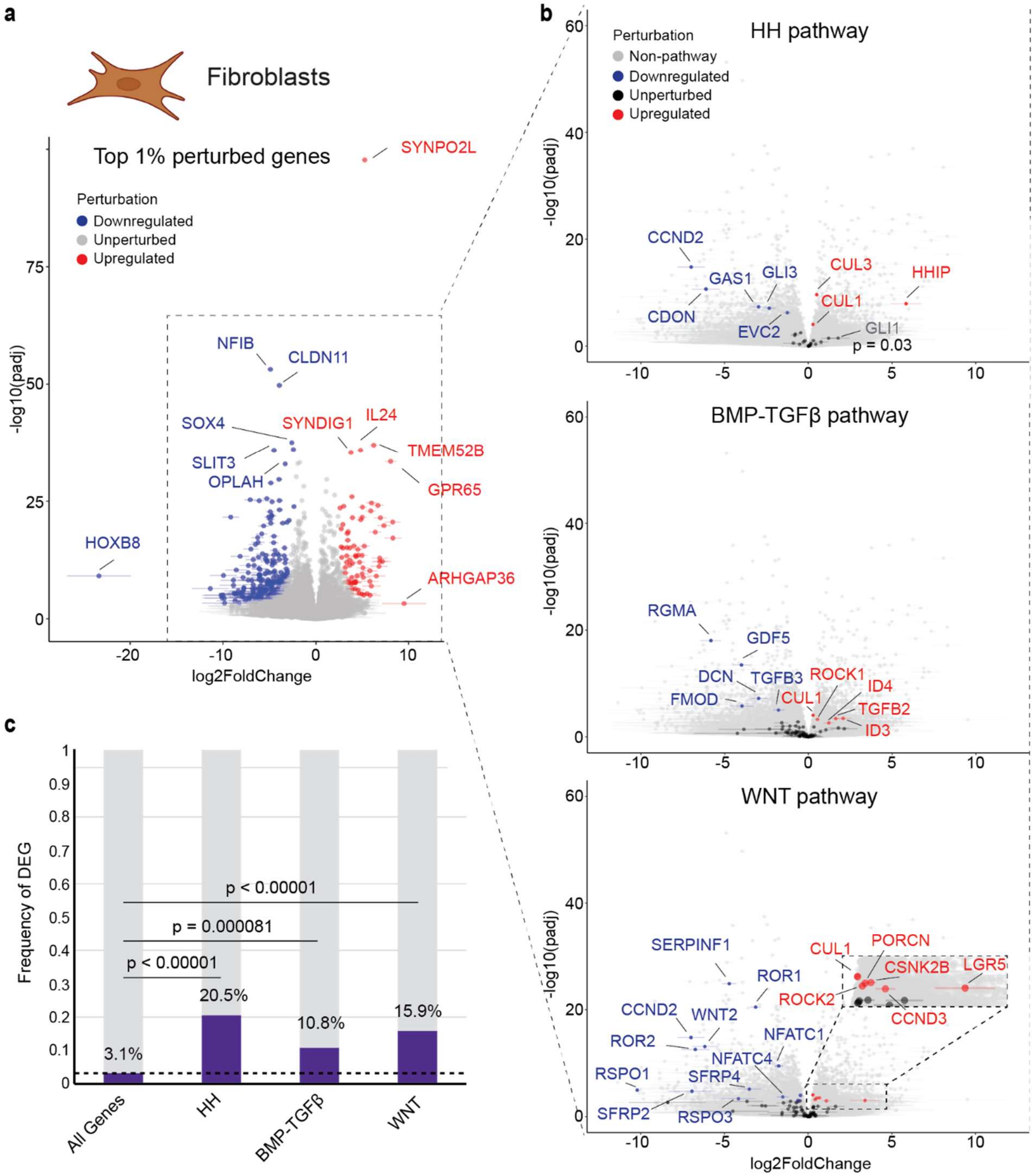

Figure S4. RNA-seq analysis from proband and control fibroblasts. (a) Volcano plot showing the top 1% of differentially expressed genes (i.e. perturbed genes) in proband fibroblast compared to matched controls. *ARHGAP36* is the gene with the highest fold change in the dataset. Statistical significance within the indicated groups was calculated using Wald test (DESeq2) and Benjamini-Hochberg multiple comparisons test with 95% confidence interval of the fitted general linear model. (b) Volcano plots of the zoomed in region highlighted by the dashed rectangle on the Figure S4A for Hedgehog, BMP-TGFβ and WNT signaling pathways. (c) Three percent of all genes were differentially expressed in the proband

compared to controls. We observed an enrichment for DEGs in HH, BMP-TGFβ and WNT signaling pathways (10-20%) in the proband. Statistical significance within the indicated groups was calculated using a chi-squared test.

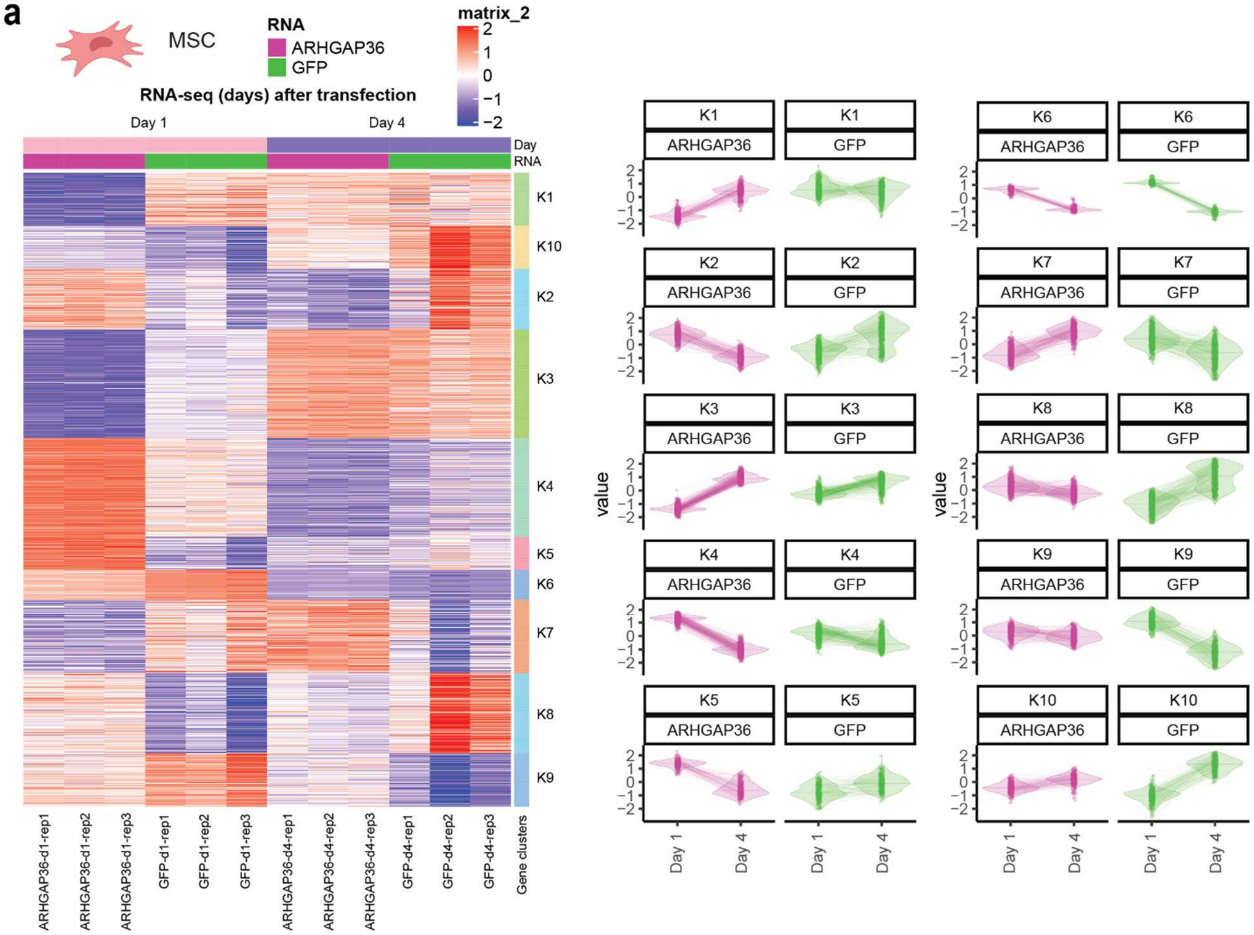

Figure S5. RNA-seq in wild type MSCs upon ARHGAP36 and GFP transient expression. (a) Ten co-expressed genes clusters (K1 to K10) were detected in the RNA-seq data of transfected samples. Among the 10 clusters, four showed high variability in ARHGAP36-transfect samples coupled with minor variance in the GFP-transfected ones (K1, K3, K4 and K5), therefore were selected for gene ontology analysis.

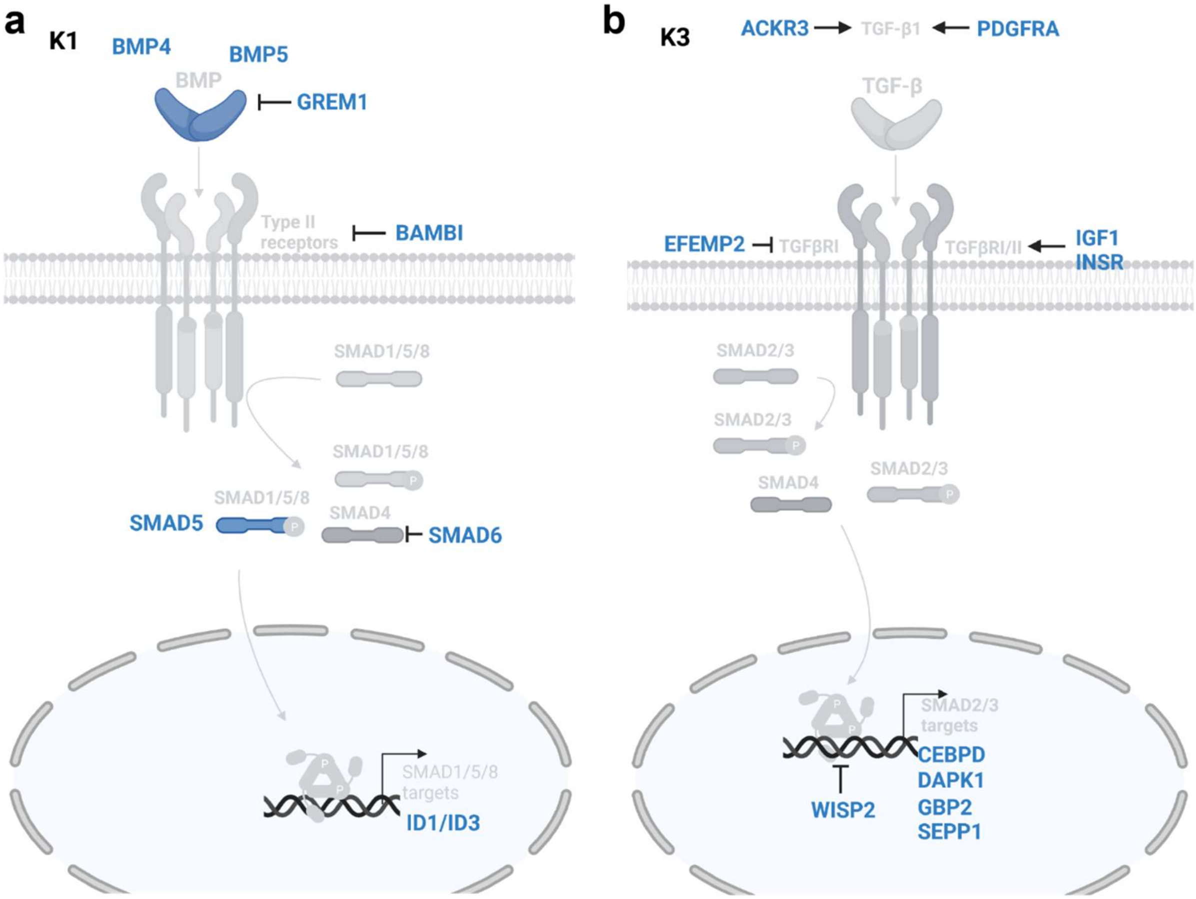

Figure S6. Co-expression clusters K1 and K3 in the BMP-TGF-β pathways. (a,b) Schematic representation of the location and interaction of key gene members of the BMP-TGFβ pathways. Non-differentially expressed genes of the pathways are shown in grey and downregulated genes are highlighted in blue.

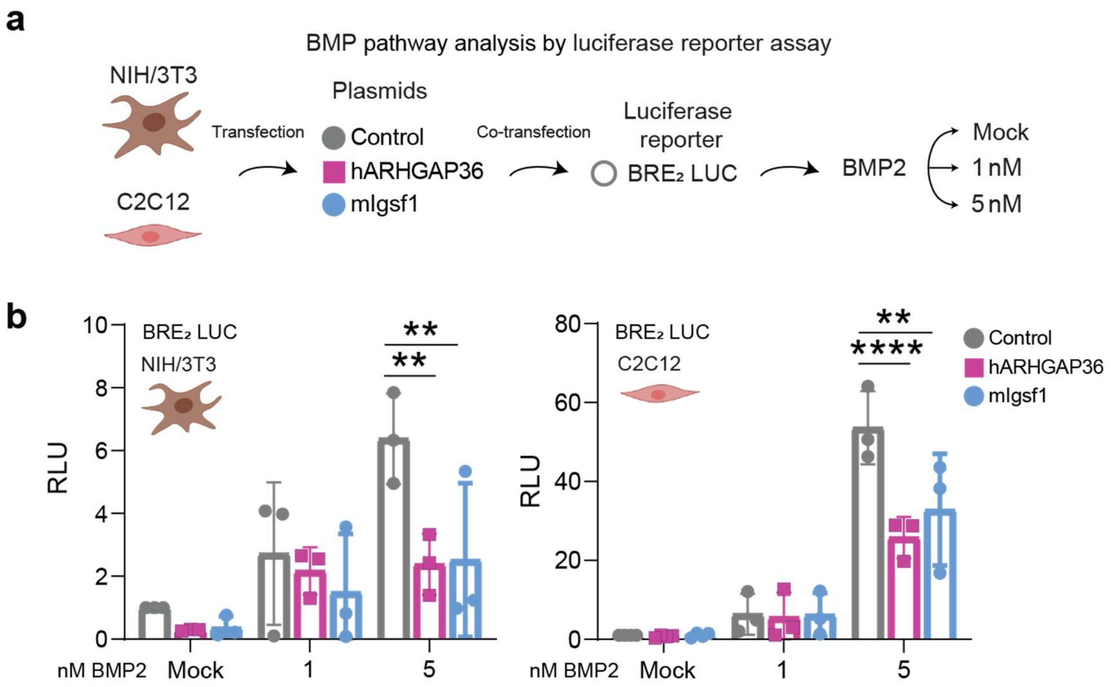

Figure S7. Human ARHGAP36 and mouse Igsf1 transient expression in mouse and human cell lines. (a) Schematics of transfection protocol in NIH/3T3 (fibroblast) and C2C12 (myoblast-like) mouse lines. Mouse cells were transfected with an empty plasmid (as control), hARHGAP36 (purple) and mIgsf1 (as positive control; green) to induce BMP pathway activity. These cells were co-transfected with BRE_2-Luc plasmid (BMP-sensitive reporter). Two different BMP2 ligand concentrations were used in this assay (1 and 5nM, plus MOCK, n=3 technical replicates. (b) Tested cells showed reduction of BMP activity only after BMP2 induction at 5 nM concentration. Statistical significance within the indicated groups was calculated using two-way ANOVA and Dunnett's multiple comparisons test; p-value: **<0.01, ****<0.001. Relative Luminescence Units (RLU) are expressed as mean fold induction ±SD over unstimulated transfected control cells.

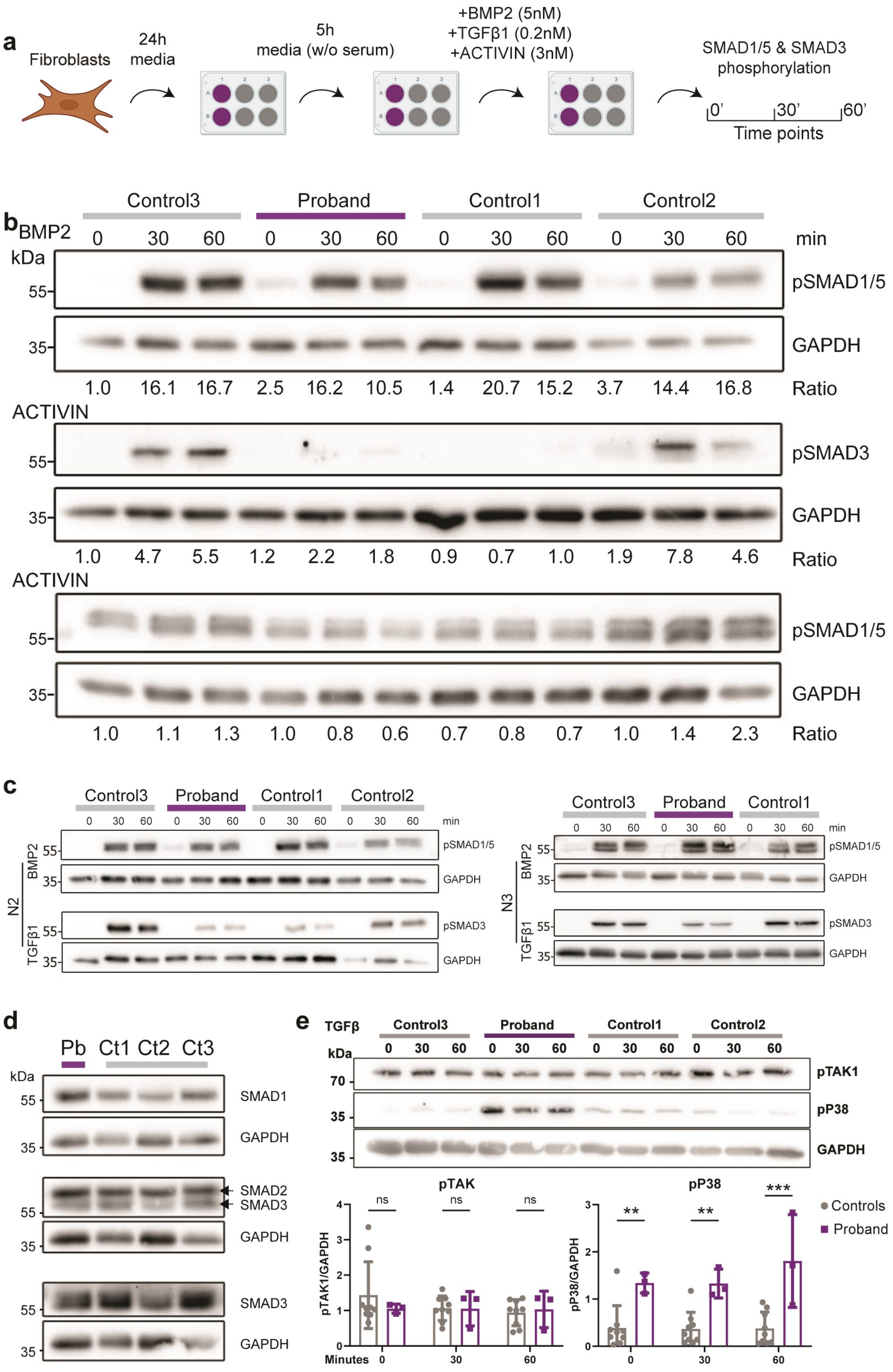

Figure S8. BMP-TGFβ pathways screening after induced by BMP2, TGFβ1 and Activin in fibroblasts. (a) Schematic representation of the experiment in proband fibroblasts and controls. Cells were

seeded for 24h in fibroblast media, the next day, media was replaced by fibroblast media without serum for 5h. Cells were induced with BMP2 (5nM), TGFβ1 (0.2nM) and Activin (3nM), and collected at three time points for SMAD1/5 and SMAD3 phosphorylation analysis via Western blot. (b) BMP pathway activity showed no difference between proband and controls. Activin showed no response in the proband sample at the three different time points, but the same pattern is observed in control 1, therefore we cannot conclude that this molecule has an impact in the studied proband. (c) Technical replicates (N2 and N3) of both BMP and TGFβ phosphorylation analysis. (d) Western blot of SMAD proteins showing no change in protein abundance in samples from proband and control. (e) Western blot of TAK1 and p38 phosphorylation levels shows difference in proband samples compared to controls in the latter. Statistical significance within the indicated groups was calculated using two-way ANOVA and Šídák's multiple comparisons test; p-value: **<0.01, ***<0.001. (n=1 proband, n=3 controls; 3 technical replicates each sample). Densitometric quantification of pTAK1 and pP38 relative to GAPDH levels expressed as mean fold induction ±SD in arbitrary units.

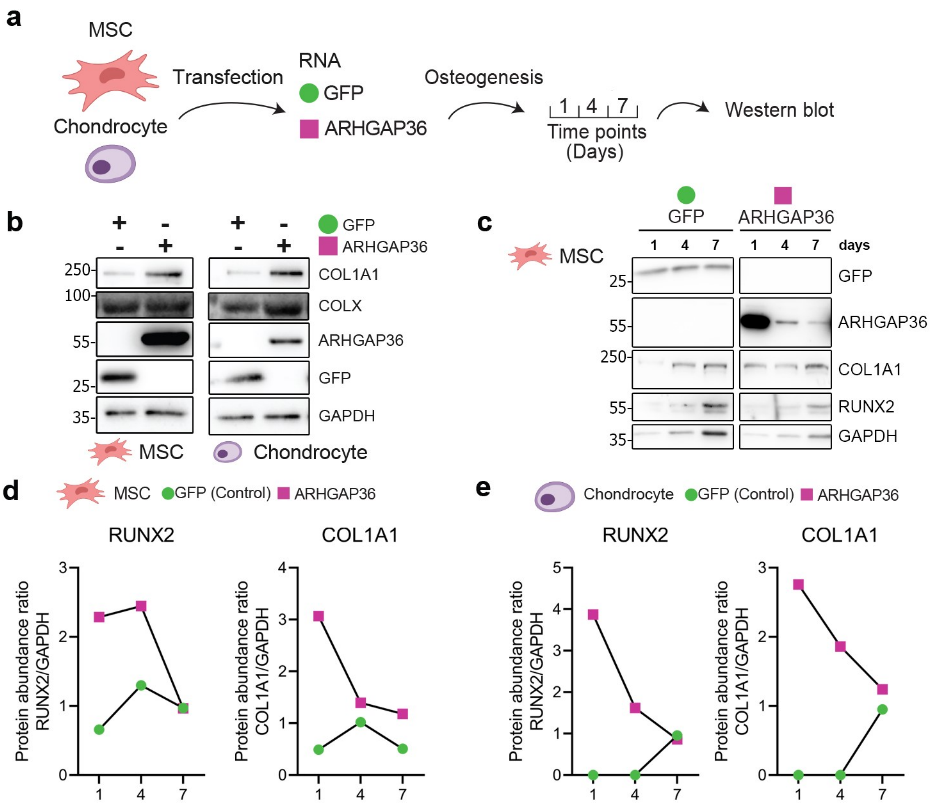

Figure S9. *ARHGAP36* transient transfection in MSCs and chondrocytes. (a) Schematics of transfection protocol of human MSCs and chondrocytes from healthy donors. Cells were transfected with *GFP* (as control; green) and *hARHGAP36* (purple) mRNA, and were exposed to osteogenic differentiation up to 7 days. (b,c) Western blot confirms ARHGAP36 and GFP transfection in both cell lines and we observed an increase of the COL1A1 and COLX ECM markers in samples transfected with hARHGAP36 than control. (d) COL1A1 and RUNX2 are upregulated in MSCs upon *ARHGAP36* transfection in the first days, reducing its expression during time. (e) Stronger early increase of ECM markers in chondrocyte cells after *ARHGAP36* transfection.

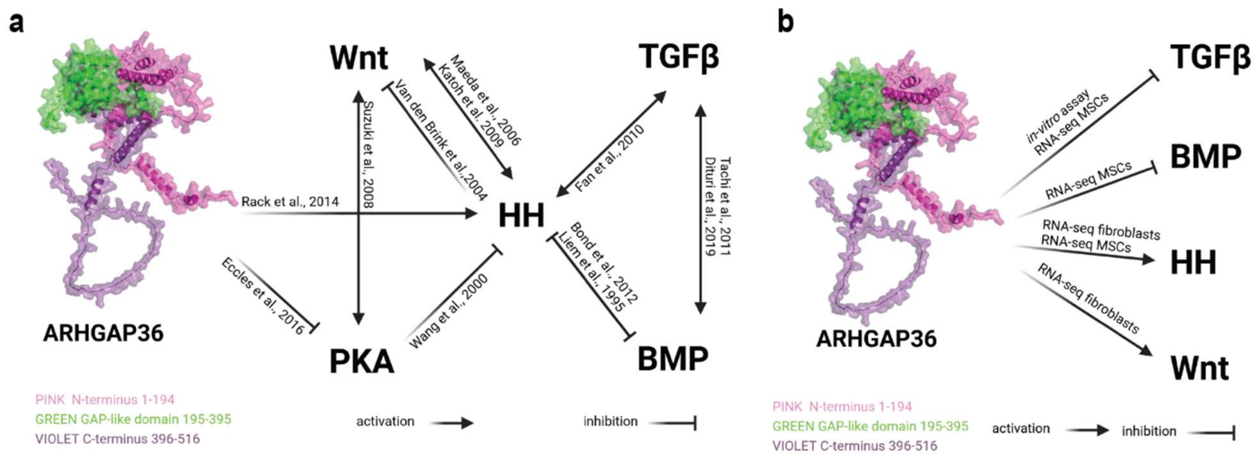

Figure 10. ARHGAP36 role in bone-related pathways. (a) Human ARHGAP36 (UniProt Q6ZRI8) structure retrieved from AlphaFold prediction. Known bone-related signaling crosstalk in the literature. ARHGAP36 is known to activate HH and inhibit PKA. (b) Outcome of ARHGAP36 overexpression in different pathways observed in this study.
